# Parasite genotype is a risk factor for *Sarcocystis neurona*-associated mortality in southern sea otters (*Enhydra lutris nereis*)

**DOI:** 10.1016/j.ijppaw.2025.101174

**Published:** 2025-12-06

**Authors:** Devinn M. Sinnott, Melissa A. Miller, Elizabeth VanWormer, Francesca Batac, Katherine Greenwald, Colleen Young, Pádraig J. Duignan, Margaret E. Martinez, Cara L. Field, Michael D. Harris, Heather Harris, Mary E. Gomes, Michael J. Murray, Karen Shapiro

**Affiliations:** a Department of Pathology, Microbiology, and Immunology, University of California, Davis School of Veterinary Medicine, 1 Shields Avenue, Davis, CA, 95616, USA; b Marine Wildlife Veterinary Care and Research Center, California Department of Fish and Wildlife, 151 McAllister Way, Santa Cruz, CA, 95060, USA; c School of Veterinary Medicine and Biomedical Sciences, School of Natural Resources, University of Nebraska-Lincoln, 1880 N 42nd Street, Lincoln, NE, 68583, USA; d The Marine Mammal Center, 2000 Bunker Road, Sausalito, CA, 94965, USA; e Monterey Bay Aquarium, 866 Cannery Row, Monterey, CA, 93940, USA

**Keywords:** Marine mammal, Sarcocystosis, California, Meningoencephalitis, Mortality event, Protozoa

## Abstract

The protozoal parasites *Sarcocystis neurona* and *Toxoplasma gondii* are important causes of mortality for threatened southern sea otters (*Enhydra lutris nereis*) in California. *Sarcocystis neurona* causes more sea otter deaths than *T. gondii* and has caused two mortality events, yet *S. neurona* is comparatively understudied. Little is known about the role of parasite genotype on the outcome of infection (fatal versus non-fatal) for *S. neurona*-infected sea otters. The objective of this study was to evaluate the effect of parasite genotype in addition to host and environmental factors (animal age, sex, location, and season of stranding) on disease outcome in *S. neurona*-infected sea otters. A multilocus sequence typing (MLST) approach was used to characterize the *S. neurona* genotype in fatal (n = 92) and non-fatal (n = 33) sea otter infections. In the northern study region, the IIg/j genotype was more likely to result in fatal infections. In the southern study region, the Ia and Ib/c/d/gg genotypes were the dominant genotypes implicated in fatal infections and were responsible for mortality events that occurred in 2004 (Ib/c/d/gg) and 2021 (Ia). Subadult sea otters were more likely to die from *S. neurona* than adults. Stranding during the California wet season during peak rainfall events that facilitate land-to-sea flow of infective sporocysts may also play a role in fatal infection outcomes. These findings suggest that parasite genotype, as well as certain host and environmental factors, all contribute to disease outcome following *S. neurona* infection in southern sea otters.

## Highlights

• *Sarcocystis neurona* genotype is a key predictor of disease outcome in sea otters.
• Ib/c/d/gg and IIg/j genotypes are more likely to be fatal in sea otters.
• Ib/c/d/gg and Ia genotypes have caused mortality events in sea otters.
• Distributions of *S. neurona* genotypes in sea otters have strong geographic patterns.

## Graphical abstract

**Figure:**
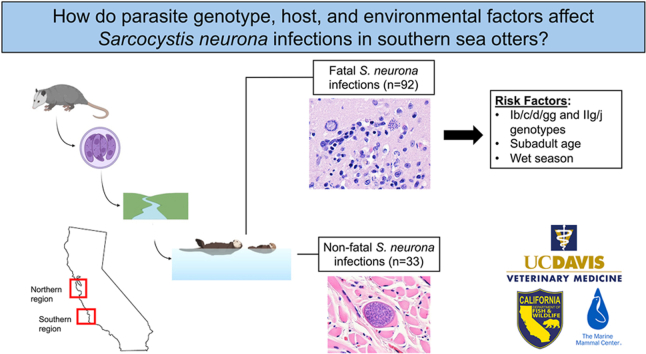

## 1 Introduction

Southern sea otters (*Enhydra lutris nereis*) are a federally protected, threatened marine mammal that serves as a keystone species in the coastal marine habitats of central California. Sea otters are commonly infected with the protozoal parasites *Sarcocystis neurona* and *Toxoplasma gondii*, with 78 % of sea otters examined between 1998 and 2012 infected with *S. neurona, T. gondii*, or both parasites concurrently (Miller et al., 2020). Although not always fatal, infection with these parasites represents a significant cause of mortality for this species, serving as a primary or contributing cause of death for 20 % of sea otters examined in the aforementioned study (Miller et al., 2020). The source of sea otter infection is land-to-sea flow of parasite-laden feces of Virginia opossums (*Didelphis virginiana*) for *S. neurona* (Shapiro et al., 2012; Burgess et al., 2020; O'Byrne et al., 2021) and wild or domestic felids for *T. gondii* (Miller et al., 2002). Despite causing more sea otter deaths than *T. gondii* (Thomas et al., 2007; Miller et al., 2020), *S. neurona* is comparatively understudied as a pathogen in this host.

*Sarcocystis neurona* causes sporadic sea otter deaths every year but unlike *T. gondii*, *S. neurona* is also capable of causing mortality events in sea otters. In April 2004, at least 40 sea otters were found dead or stranded alive but were later euthanized or died over a four-week period in Estero Bay, California (Miller et al., 2010). This episode represented the largest localized mortality event ever recorded in over 50 years of sea otter stranding data collection. Infection with *S. neurona* was implicated as the cause of this mortality event, as affected animals had histopathologic evidence of systemic sarcocystosis characterized by moderate to severe meningoencephalitis, myocarditis, and/or myositis and *S. neurona* DNA was amplified from affected tissues. A second *S. neurona*-associated mortality event in Estero Bay occurred in April 2021 resulting in 30 confirmed deaths. These mortality events can have important impacts on the southern sea otter population which is struggling to return to its historic population size and geographical range. Although several factors were hypothesized to contribute to the 2004 mortality event, much remains unknown. Heavy rainfall prior to the start of that event may have led to increased surface water runoff carrying infective stages of *S. neurona* (sporocysts) to near-shore waters (Miller et al., 2010). Another potential risk factor is peak southern sea otter pupping in late winter (Chinn et al., 2016), resulting in higher numbers of especially vulnerable young animals during spring storm events with maximal land-to-sea parasite flow.

While host and environmental factors have been postulated to mediate *S. neurona* infections and mortality in sea otters, the role of pathogen virulence has been largely unexplored. Differences in virulence and host immune response to infection have been demonstrated among different strains of *T. gondii* in animal models (Sibley and Boothroyd, 1992; Melo et al., 2011; Su et al., 2012). In southern sea otters, only Type X, X variants, and the COUG strains of *T. gondii* have been implicated in fatal toxoplasmosis (Shapiro et al., 2019; Miller et al., 2023). It is possible that *S. neurona* may exhibit strain-specific virulence similar to that of *T. gondii* that could trigger more severe disease and mortality events in sea otters. Previous studies have reported diverse *S. neurona* genotypes infecting sea otters (Wendte et al., 2010a, 2010b; Rejmanek et al., 2010; Barbosa et al., 2015). However, no reports to date have assessed *S. neurona* genotype in relation to lesion severity or the outcome of infection (e.g., survival or death) with the exception that animals stranding with fatal sarcocystosis during the 2004 mortality event were all reported to be infected with the same group of closely related *S. neurona* genotypes (Wendte et al., 2010a, 2010b; Rejmanek et al., 2010). The goal of this study was to assess associations between disease outcome (fatal versus non-fatal infection) and *S. neurona* genotypes as well as specific host and environmental factors.

## 2 Materials and methods

### 2.1 Study animals and ethics statement

This study included: a) southern sea otter carcasses that were found dead along the California coast and recovered by the California Department of Fish and Wildlife (CDFW), The Marine Mammal Center (TMMC), Monterey Bay Aquarium (MBA), or other authorized members of the Southern Sea Otter Stranding Network (SSOSN); b) sea otters that stranded moribund and were subsequently humanely euthanized by CDFW, TMMC, MBA, or other SSOSN staff under standardized animal care protocols and veterinary oversight; and c) sea otters that died naturally or were humanely euthanized due to poor prognosis during rehabilitation efforts by veterinary staff at TMMC or the MBA. A gross necropsy was performed for all sea otters by veterinary pathologists and necropsy staff at CDFW and TMMC. Demographic data including age class (immature [6 months-1 year], subadult [1–3 years], adult [4+ years]), sex, and stranding date and location were recorded. Age class categories were determined based on dentition as previously described (Kreuder et al., 2003; Miller et al., 2020). The recovery of carcasses, humane euthanasia of sea otters, and sample collection by CDFW were all performed in accordance with Section 109(h) of the U.S. Marine Mammal Protection Act (MMPA) and the U.S. Fish and Wildlife Service (USFWS) regulations implementing the MMPA at 50 CFR 18.22(a), and in accordance with the Service's regulations implementing the U.S. Endangered Species Act at 50 CFR 17.21(c)(3). Permission for animal or carcass recovery, rehabilitation, humane euthanasia, and sample collection was granted by USFWS permits MA101713-1 and PER4324646 for TMMC, and MA032027 and MA186914 for MBA.

### 2.2 Histopathology and cause of death determination

Tissues selected for histopathologic examination varied depending on the postmortem condition of individual sea otters. At minimum, a partial set of tissues including brain, heart, tongue, liver, lung, and spleen were examined microscopically for all sea otters in this study. For euthanized cases or animals found dead within a short postmortem interval, a more extensive set of tissues was examined including the minimum tissues listed above as well as kidney, endocrine organs, the gastrointestinal and reproductive tracts, skeletal muscles, and other tissues. Tissues were fixed in 10 % neutral buffered formalin, trimmed, embedded in paraffin, sectioned at 5 μm thickness, and stained with hematoxylin and eosin (H&E). Histopathology slides were reviewed by 1–2 veterinary pathologists (DMS, MAM, PJD, MEM) for evidence of *S. neurona* infection, with particular examination of brain, heart, and skeletal muscle, as these organs are commonly parasitized by *S. neurona* in infected sea otters (Miller et al., 2009). Features examined included: 1) the presence and relative burden (low, medium, high) of various *S. neurona* stages (merozoites, schizonts, sarcocysts) in affected tissues, and 2) the presence, relative severity (mild, moderate, severe), and type (lymphoplasmacytic, pleocellular, etc.) of inflammation associated with protozoal organisms in affected tissues. Immunohistochemistry (performed at the California Animal Health and Food Safety Laboratory, Davis, CA) was used to definitively identify *S. neurona* in tissues as needed. Evidence of comorbidities or other concurrent disease processes were also noted. Based on gross examination, histopathologic findings, and immunohistochemistry, each sea otter was assigned up to four causes of death: one primary cause of death and up to three contributing causes of death. The primary cause of death was the disease process that was most severe and immediately life-threatening to the animal, or most likely to have warranted humane euthanasia. Contributing causes of death were other independent disease processes that were moderate to severe in nature at the time of death and likely resulted in clinically significant disease that contributed to each animal's death.

### 2.3 Case and control definitions and selection

For this study, sea otters were categorized as having fatal *S. neurona* infections if sarcocystosis was determined to be a primary or contributing cause of death. Sea otters were categorized as non-fatal *S. neurona* infections if there was evidence of *S. neurona* infection (e.g., quiescent sarcocysts observed in skeletal muscle unassociated with inflammation) but sarcocystosis was not determined to be a primary or contributing cause of death. Previous studies described molecular characterization and genotyping of *S. neurona* from 57 sea otters sampled from 1999 to 2008, encompassing 55 fatal infections (including 12 sea otters that died during the 2004 mortality event (Wendte et al., 2010b, 2010a; Rejmanek et al., 2010)) and 2 non-fatal infections. An additional 37 fatal cases were molecularly characterized in this study, including 12 sea otters that died during the 2021 mortality event and 25 opportunistically collected carcasses from 2022 to 2023. An additional 31 non-fatal infections (controls) occurring during an overlapping time period (1998–2012) were selected from a dataset published by Miller et al. (2020), as well as opportunistically collected carcasses in 2021.

### 2.4 Parasite isolation

Parasites were isolated in culture from brain tissue for all previously genotyped cases (n = 57) (Wendte et al., 2010a, 2010b; Rejmanek et al., 2010). For a subset of the opportunistically sampled sea otters collected in 2022 and 2023 that were euthanized or freshly deceased at the time of necropsy (n = 9), isolation of *S. neurona* from brain tissue was performed. For each of these cases, brain tissue was collected aseptically and placed in saline supplemented with penicillin/streptomycin and amphotericin B for at least 24 h. Brain tissue was then homogenized and approximately 1 ml of homogenate was added to a flask of MA-104 monkey kidney cells. The flask was incubated at 37 °C and 5 % CO_2_ for 2 h, after which the flask was rinsed with RPMI media to remove the homogenate. Cultures were maintained at 37 °C and 5 % CO_2_ for 30 days and monitored three times per week for signs of parasite growth, at which time 5 ml of fresh RPMI media was exchanged. Once parasite growth was established, the supernatant containing extracellular parasites was collected and centrifuged at 13,000 rpm for 10 min to pellet merozoites for subsequent DNA extraction.

### 2.5 DNA extraction and polymerase chain reaction (PCR) at the ITS1 locus

For fatal *S. neurona* infections, DNA was extracted from frozen brain tissue, cultured merozoites, or frozen subcutaneous adipose tissue for one case with grossly apparent steatitis. For non-fatal *S. neurona* infections, DNA was extracted from frozen brain and tongue in triplicate and screened for parasite DNA via amplification at the ITS1 locus for an initial subset of ten non-fatally infected sea otters. Tongue proved to be the most sensitive tissue for detecting *S. neurona* DNA from non-fatal infections in this initial trial (n = 7/10 positive for tongue versus n = 1/10 positive for brain). As a result, tongue was used for DNA extraction and PCR for the remaining samples from sea otters with non-fatal infections. For both fatal and non-fatal infections, DNA was extracted in triplicate from the selected tissue sample using a DNeasy Blood and Tissue kit (Qiagen, Valencia, CA) according to the manufacturer's protocol with one modification: 180 μl ATL buffer and 30 μl proteinase K were added to each sample and incubated at 56 °C overnight.

To confirm that *S. neurona* DNA was present, extracted DNA from each sea otter was screened using a nested PCR assay targeting the multi-copy ITS1 locus using previously described pan-apicomplexan primers (Rejmanek et al., 2009). Each external PCR reaction contained 22 μl nuclease-free water, 25 μl AmpliTaq Gold master mix (Applied Biosystems, Foster City, CA), 0.5 μl of each external primer (50 μM), and 2 μl DNA for a total reaction volume of 50 μl. Internal PCR reactions contained 23 μl nuclease-free water, 25 μl AmpliTaq Gold master mix, 0.5 μl of each internal primer (50 μM), and 1 μl product of the external reaction for a total reaction volume of 50 μl. Nuclease-free water was used as a negative control and DNA extracted from pelleted *T. gondii* tachyzoites grown in culture from a sea otter brain was used as a positive control for each assay. Primers and cycling conditions for each reaction are listed in Supplemental Table 1.

Each internal PCR product was electrophoresed on a 2 % agarose gel stained with RedSafe (Bulldog Bio, Portsmouth, NH). Amplified bands were excised and purified using a QIAquick gel extraction kit (Qiagen, Valencia, CA) according to the manufacturer's protocol. Purified amplicons were submitted for sequencing in the forward and reverse directions (UCDNA Sequencing Facility, Davis, CA or Genewiz, South San Francisco, CA). Forward and reverse sequence chromatograms were aligned, trimmed, and evaluated for base call quality using Geneious (Dotmatics, Auckland, New Zealand). Consensus sequences obtained from alignment of forward and reverse sequences were compared to a reference database in GenBank using BLASTn (https://blast.ncbi.nlm.nih.gov/Blast.cgi). Samples yielding sequences with >99 % identity to *S. neurona* reference sequences in GenBank were further characterized using multilocus sequence typing (MLST).

### 2.6 Multilocus sequence typing (MLST)

The *S. neurona* genotype causing each fatal and non-fatal infection was determined using a nested or hemi-nested conventional PCR-based multilocus sequence typing (MLST) approach targeting six loci: three *S. neurona* surface antigens (SnSAG1-5-6, SnSAG3, and SnSAG4) and three *S. neurona* microsatellite loci (sn3, sn7, sn9). These loci were selected as they have been previously demonstrated to exhibit moderate to high polymorphism across *S. neurona* genotypes (SnSAG3, SnSAG4, sn3, sn7, sn9) (Rejmanek et al., 2010; O'Byrne et al., 2021) or represent paralogous genes that are variably expressed across genotypes (SnSAG1-5-6) (Howe et al., 2008; Crowdus et al., 2008; Wendte et al., 2010b). For each sea otter, one DNA replicate that was confirmed to contain *S. neurona* DNA via screening at the ITS1 locus was further characterized at these six MLST loci as previously described (Asmundsson and Rosenthal, 2006; Wendte et al., 2010b; Rejmanek et al., 2010). Each external PCR reaction contained 22 μl nuclease-free water, 25 μl AmpliTaq Gold master mix, 0.5 μl each of external forward and reverse primers (50 μM), and 2 μl DNA for a total of 50 μl. Each internal PCR reaction contained 23 μl nuclease-free water, 25 μl AmpliTaq Gold master mix, 0.5 μl each of internal forward and reverse primers (50 μM), and 1 μl product from the external reaction for a total of 50 μl. Additional internal forward and reverse primers were designed to create a nested assay for the SnSAG1-5-6 locus to increase sensitivity of amplification. Nuclease-free water was used as a negative control and DNA extracted from pelleted *S. neurona* merozoites and schizonts grown in culture from a harbor seal brain was used as a positive control for each assay. Primers and cycling conditions for each reaction are listed in Supplemental Table 1.

Following amplification, each sample underwent gel electrophoresis, DNA purification, sequencing, and chromatogram alignment and trimming as described in Section 2.5. For the SnSAG1-5-6 locus, trimmed consensus sequences were compared to reference sequences in GenBank using BLAST to confirm their identity as either SnSAG1, SnSAG5, or SnSAG6 based on the highest percent identity to published reference sequences. For the SnSAG3 locus, trimmed consensus sequences were aligned to a reference sequence (GenBank GQ851954, amplified from a sea otter-derived *S. neurona* isolate) and single nucleotide polymorphisms (SNPs) were noted at nucleotide positions 239, 503, 504, and 1057. For the SnSAG4 locus, trimmed consensus sequences were aligned to a reference sequence (GenBank GQ851957, amplified from a sea otter-derived *S. neurona* isolate) and SNPs were noted at nucleotide position 592. For the microsatellite loci, the number of dinucleotide repeats (AT for sn3, CA for sn7, and GT for sn9) were counted directly from the chromatogram. Each sample was then assigned a genotype including an antigen type (designated as a Roman numeral) based on the pattern of SNPs across the SnSAG loci and a microsatellite type (designated as a lowercase letter) based on the number of dinucleotide repeats at each locus as previously described (Wendte et al., 2010a; Barbosa et al., 2015).

### 2.7 Stranding season classification

The California climate is characterized by a hot, dry season (May–September) and a mild, wet season (October–April), with an average of 75 % of the state's annual precipitation falling between November and March, regardless of whether a given year is relatively wet or dry (Milanes et al., 2022). The classification of stranding season for each sea otter was established based on these local precipitation patterns and adjusted to account for the time required after precipitation events for *S. neurona* sporocysts to travel in freshwater surface runoff into the marine environment and for sea otters to become infected (Shapiro et al., 2012). We therefore categorized the “adjusted wet season” based on prior work demonstrating a 30–60-day lag between peak coastal streamflow following rainfall and subsequent *S. neurona* sea otter mortalities in central California (Shapiro et al., 2012). Specifically, a sea otter was determined to strand during the wet season if stranding occurred within the interval beginning one month after heavy precipitation typically begins (December). The end of the wet season interval was extended two months from March to May to account for the time required for sea otters to become infected and develop clinical sarcocystosis, which can range from approximately 1 to 2 months (Miller et al., 2010; Shapiro et al., 2012). Thus, sea otters were considered to have stranded in the wet season if the date of stranding fell within the adjusted wet season interval (December–May), or were considered to have stranded in the dry season if the date of stranding fell outside the adjusted wet season interval (June–November).

### 2.8 Spatial analysis

Latitudinal and longitudinal coordinates of stranding location were assigned for each sea otter. Coordinates for more recent cases were recorded at the time of collection with a GPS unit or phone-based mapping application, or estimated using a mapping program. For older cases, coordinates were calculated by converting As-The-Otter-Swims (ATOS) points, a pre-GPS system which consists of a 1-dimensional axis with points spaced at 500-m-long intervals along the entire California coastline at the 5-fathom depth contour, to cartesian coordinates. The geographic location of each sea otter and their respective *S. neurona* genotype was mapped using qGIS (version 3.34.2-Prizren). Sea otters were categorized into two geographic regions (northern and southern) based on their stranding location relative to latitude 35.89°N (Cape San Martin, the approximate center of the southern sea otter range).

### 2.9 Data analysis

The prevalence of each *S. neurona* genotype was calculated for the overall study population, as well as within the fatal and non-fatal infection groups and within each geographic region (northern and southern). Univariable bias-reduced logistic regression models were performed for each demographic or environmental variable (genotype, age, sex, region, stranding season) to assess associations with *S. neurona* infection outcome. Due to spatial differences in the regional distribution of *S. neurona* genotypes, logistic regression models were initially performed for three datasets: 1) all sampled sea otters (adult, subadult, and immature animals; n = 125); 2) all sampled sea otters in the northern region (n = 63); and 3) all sampled sea otters in the southern region (n = 62). Since immature sea otters were excluded from the 1998–2012 dataset from which non-fatal infections were selected (Miller et al., 2020), models were also run with reduced datasets restricted to adult and subadult sea otters in the overall population (n = 91), the northern region (n = 52), and the southern region (n = 39).

Variables with a p-value <0.20 in univariable models were evaluated in multivariable bias-reduced logistic regression models. Using a purposeful selection model-building approach (Hosmer and Lemeshow, 2000), variables were retained in the model when p ≤ 0.05. Sex, which was significantly associated with *S. neurona* infection and mortality in previous studies (Johnson et al., 2009; Burgess et al., 2020; Miller et al., 2020), was also assessed as a potential confounding variable in multivariable models. Akaike's information criterion was used to select a parsimonious multivariable model for each dataset. Regression analyses were performed using the brglm package (Kosmidis, 2023) in R v. 4.4.0 (R Core Team, 2024).

## 3 Results

### 3.1 Study population

Based on findings from gross examination, histopathology, and immunohistochemistry, 92 sea otters were identified with fatal infections. Sarcocystosis was determined to be the primary cause of death for nearly all fatal cases (n = 85/92), and served as a secondary, tertiary, or quaternary cause of death for a small subset of fatal cases (n = 7/92). Fatal infections had histopathologic evidence of moderate to severe meningoencephalitis often with intralesional merozoites and schizonts as has been previously described in sea otters infected with this parasite (Miller et al., 2010, 2018; Thomas et al., 2007). Many of these sea otters had other comorbidities at the time of death. While a detailed list of the concurrent disease processes diagnosed in this large group of animals is beyond the scope of the present study, previous studies have described the lesions and epidemiology of common diseases affecting southern sea otters (Kreuder et al., 2003; Miller et al., 2018, 2020). The presence of *S. neurona* DNA was confirmed in frozen brain tissue (n = 27), frozen subcutaneous adipose tissue (n = 1), or parasites isolated from brain in culture (n = 64) via screening at the ITS1 locus for each sea otter with fatal sarcocystosis. The *S. neurona* strains from 55 of the 92 fatal infections were genotyped in previous studies (Wendte et al., 2010a, 2010b; Rejmanek et al., 2010), while 37 were genotyped during the present study. A total of 126 sea otters with non-fatal *S. neurona* infections were also identified. Non-fatal infections were characterized histopathologically by the presence of intramuscular sarcocysts unassociated with inflammation in skeletal muscles as has been previously described (Miller et al., 2018). Primary causes of death for non-fatal infections included: shark bite trauma (n = 11), cardiomyopathy (n = 7), domoic acid intoxication (n = 3), conspecific fight trauma (n = 3), boat strike (n = 2), bacterial infections (n = 2), enterocolitis (n = 2), end lactation syndrome (n = 1), viral infection (n = 1), and undetermined (n = 1). For these chronic, non-fatal *S. neurona* infections which typically feature very low numbers of mature sarcocysts in skeletal muscle, DNA was extracted from frozen tongue in triplicate and screened at the ITS1 locus. Samples from 41/126 non-fatal infections yielded a band consistent in size with *S. neurona* with the ITS1 assay and were genotyped with MLST. A complete (6/6 loci) or near complete (5/6 loci) *S. neurona* genotype was successfully characterized for 31/41 sea otters. Parasite strains from two additional non-fatal infections were genotyped in previous studies using isolates from brain in culture (Wendte et al., 2010a, 2010b; Rejmanek et al., 2010). By combining previously published and newly generated data, genotypes were characterized from a total of 125 sea otters including 92 animals with fatal *S. neurona* infections *and 33 non-fatal S. neurona infections* (Supplemental Table 2)*.*

*Toxoplasma gondii* DNA was co-amplified by the ITS1 assay for a subset of these 125 sea otters. However, systematic testing for *T. gondii* co-infection (i.e. performing PCR on multiple tissues and using *T. gondii*-specific PCR assays when *S. neurona* DNA may have been preferentially amplified) was not performed for most cases in the present study and the definitive co-infection status for all sea otters was not determined. Therefore, co-infection with *T. gondii* was not included as a variable in logistic regression models.

### 3.2 Distribution of *S. neurona* genotypes

**Fig. 1:**
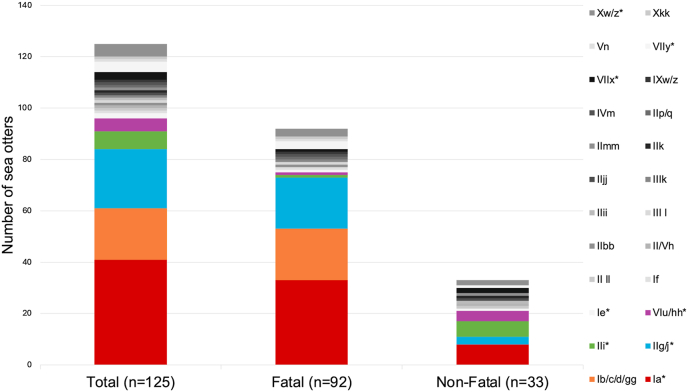
Distribution of *Sarcocystis neurona* genotypes identified in the total study population (n = 125), fatal *S. neurona* infections (n = 92), and non-fatal *S. neurona* infections (n = 33). Asterisks indicate eight genotypes that were present in both the fatal and non-fatal disease outcome groups.

Twenty-four *S. neurona* genotypes (unique combinations of antigen and microsatellite types) were identified in these southern sea otters (Fig. 1). Microsatellite types that could not be distinguished based on the three selected microsatellite loci were collapsed (e.g., b/c/d/gg, g/j, etc.). Antigen types that could not be distinguished due to lack of amplification at one SnSAG locus were similarly collapsed (e.g., II/V). The most prevalent genotypes in the overall study population included Ia (n = 41, 32.8 %), IIg/j (n = 23, 18.4 %), and Ib/c/d/gg (n = 20, 16 %). All other genotypes had less than 10 sea otters represented, with individual prevalences of <10 % (Supplemental Table 3). Of the 24 identified *S. neurona* genotypes, 10 were exclusively found in the fatal infection group, six were exclusively found in the non-fatal infection group, and eight genotypes overlapped between both disease outcome groups (Fig. 1).

**Fig. 2:**
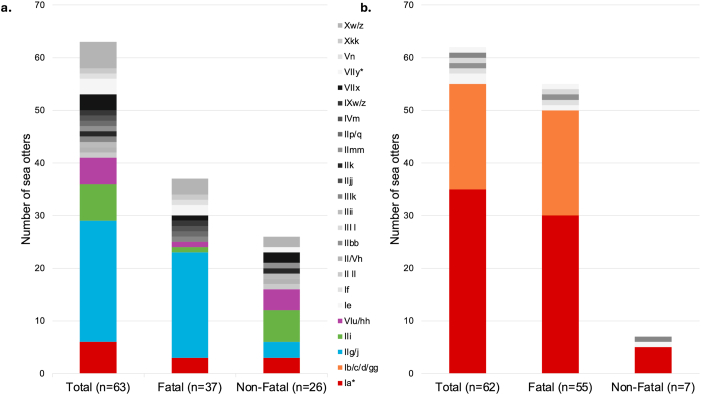
Distribution of *Sarcocystis neurona* genotypes for regional subpopulations and fatal and non-fatal disease outcome groups in the northern region (a) and southern region (b). Asterisks indicate genotypes present in both the northern and southern regions.

**Fig. 3:**
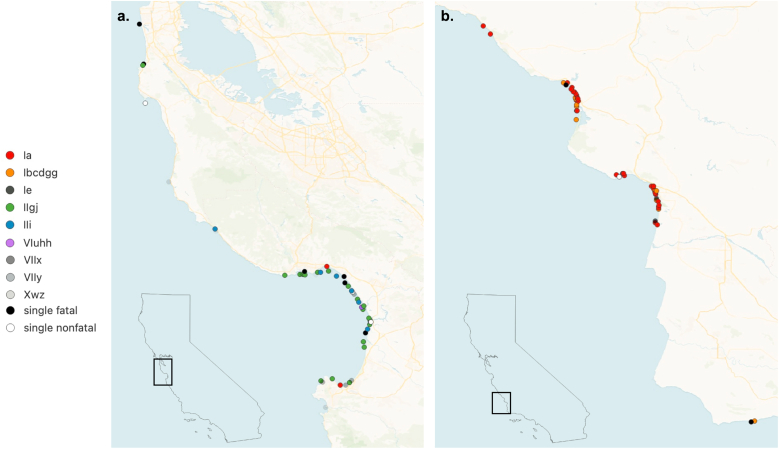
Geographic distribution of the most common *Sarcocystis neurona* genotypes by individual sea otter stranding location in the northern (a) and southern (b) study regions. Genotypes represented by a single infected sea otter were collapsed by disease outcome into “single fatal” and “single non-fatal” groups. Base map and map data from © OpenStreetMap and OpenStreetMap Foundation. https://www.openstreetmap.org/copyright.

Distinct geographic patterns emerged for *S. neurona* genotypes identified in fatal and non-fatal infections. Eighteen genotypes were identified in sea otters that stranded in the northern study region (n = 63), including 16 genotypes that were only found in this region (Fig. 2, Fig. 3). The IIg/j genotype was exclusively identified in the northern region and represented the most prevalent genotype in this area (n = 23/63, 36.5 %). This genotype accounted for 54.1 % of fatal infections (n = 20/37) in sea otters from the northern region (Fig. 2a) with relatively even distribution over time (Supplemental Fig. 1). Two other genotypes that were exclusively found in the northern region, IIi (n = 7/63, 11.1 %) and VIu/hh (n = 5/63, 7.9 %), were the most common genotypes identified in non-fatal infections in this region (Fig. 2a).

Eight *S. neurona* genotypes were identified in sea otters that stranded in the southern study region (n = 62), six of which were only found in this region (Fig. 2, Fig. 3). The Ia genotype accounted for the majority of overall *S. neurona* infections (n = 35/62, 56.5 %), including the majority of fatal (n = 30/55, 54.5 %) and non-fatal infections (n = 5/7, 71.4 %) in the southern region (Fig. 2b). Additionally, all 12 of the sea otters characterized from the 2021 *S. neurona*-associated mortality event were infected with the Ia genotype. The Ib/c/d/gg genotype was exclusively found in the southern region, where it was the second most prevalent genotype (n = 20/62, 32.3 %) (Fig. 2b). All sea otters infected with the Ib/c/d/gg genotype died from sarcocystosis, and this genotype accounted for 36.5 % (n = 20/55) of fatal infections in the southern region, including all 12 of the sea otters characterized from the 2004 *S. neurona*-associated mortality event (Wendte et al., 2010a). The Ia and Ib/c/d/gg genotypes had an inverse temporal distribution in the southern region, with the Ib/c/d/gg genotype more commonly observed in fatal infections prior to 2021, and the Ia genotype appearing as the dominant genotype in fatal infections since at least 2021 (Supplemental Fig. 1).

### 3.3 Associations between fatal sarcocystosis and parasite, host, and environmental risk factors

The southern study region had a greater proportion of fatal infections (n = 55/62, 88.7 %) compared to the northern region (n = 37/63, 58.7 %). In univariable models of the total study population, sea otters stranding in the southern region had significantly higher odds of fatal *S. neurona* infection than sea otters in the northern region (Table 1, Supplemental Table 5). Due to the exclusive presence of many genotypes in either the northern or southern study regions, regionally specific univariable and multivariable regression models were performed as well as models for the overall study population combining animals from both regions. Additionally, all immature sea otters included in this study (n = 33) represented fatal infections (cases) since immature sea otters were excluded from the archival dataset from which non-fatal infections (controls) were selected (Miller et al., 2020). This lack of an appropriate control for the immature age group limits the ability to draw meaningful conclusions for this age class. To account for this, primary analyses focused on a reduced total population and regional subpopulations that excluded immature sea otters (Table 1, Table 2, Supplemental Table 4). For comparison, univariable and multivariable models were also performed for the entire population and regional subpopulations including immature sea otters (Supplemental Tables 5 and 6) with similar trends observed. Variables significantly associated with fatal *S. neurona* infection in univariable and multivariable models for the reduced total population and regional subpopulations included genotype (IIg/j in the north, and Ib/c/d/gg in the south) and age class (subadult) (Table 1, Table 2). Stranding during the wet season was significantly associated with fatal *S. neurona* infection in univariable models of the overall population and regional populations without immature sea otters (Table 1) and was marginally significant in its association with fatal infection in the multivariable model for the northern region (Table 2). Including stranding season in the final multivariable models enhanced model fit for the northern region but not for the southern region. Sex was not significantly associated with fatal *S. neurona* infection in any models, and no confounding variables were identified.

**Table 1:** Univariable bias-reduced logistic regression results for associations between fatal *S. neurona* infections and parasite (genotype, evaluated as binary variables [i.e. a specific genotype vs. all other genotypes] and categorical variables), host (age, sex) and environmental (season, geographic region) risk factors analyzed for the entire study population and regional subpopulations with immature otters excluded. Variables with p-values ≤0.2 (bolded) were evaluated further in multivariable models. OR, odds ratio; REF, reference category.

Variable	Level	Total Population (n = 91)		Northern Population (n = 52)		Southern Population (n = 39)	
		OR	p-value	OR	p-value	OR	p-value
*Genotype Ia*							
	Other genotypes	1.00	REF	1.00	REF	1.00	REF
	Ia	1.07	0.90	1.00	1.00	**0.28**	**0.14**

*Genotype Ib/c/d/gg*							
	Other genotypes	1.00	REF	1.00	REF	1.00	REF
	Ib/c/d/gg	**30.06**	**0.02**	–	–	**1.94**	**0.06**

*Genotype IIg/j*							
	Other genotypes	1.00	REF	1.00	REF	1.00	REF
	IIg/j	**2.59**	**0.15**	**6.71**	**0.01**	–	–

*Genotype VIu/hh*							
	Other genotypes	1.00	REF	1.00	REF	1.00	REF
	VIu/hh	**0.17**	**0.10**	0.29	0.25	–	–

*Genotype IIi*							
	Other genotypes	1.00	REF	1.00	REF	1.00	REF
	IIi	**0.11**	**0.03**	**0.19**	**0.10**	–	–

*Genotype (categorical)*							
	Other genotypes	1.00	REF	1.00	REF	1.00	REF
	Ia	2.17	0.21	1.24	0.83	2.27	0.47
	Ib/c/d/gg	**44.05**	**0.01**	–	–	**37.00**	**0.04**
	IIg/j	**4.59**	**0.04**	**4.76**	**0.05**	–	–
	VIu/hh	0.40	0.41	0.41	0.43	–	–
	IIi	0.27	0.22	0.29	0.24	–	–

*Sex*							
	Male	1.00	REF	1.00	REF	1.00	REF
	Female	1.60	0.34	1.80	0.34	0.78	0.77

*Age*							
	Adult	1.00	REF	1.00	REF	1.00	REF
	Subadult	**10.64**	**<0.01**	**6.74**	**<0.01**	**28.04**	**0.03**

*Season*							
	Dry	1.00	REF	1.00	REF	1.00	REF
	Wet	**7.28**	**<0.01**	**5.80**	**<0.01**	**7.28**	**<0.01**

*Geographic region*							
	Northern	1.00	REF	–	–	–	–
	Southern	**4.33**	**<0.01**	–	–	–	–

**Table 2:** Multivariable bias-reduced logistic regression results for associations between fatal *S. neurona* infections and parasite genotype (evaluated as a binary variable), age class, and stranding season analyzed for each regional subpopulation excluding immature otters. Stranding season was excluded from the southern population model as it was not statistically significant and did not improve model fit. OR, adjusted odds ratio; CI [95 %], 95 % confidence interval, REF, reference category.

Variable	Level	Northern Population (n = 52)			Southern Population (n = 39)		
		OR	CI [95 %]	p-value	OR	CI [95 %]	p-value
*Genotype*							
	Other genotypes	1.00	REF	REF	1.00	REF	REF
	Ib/c/d/gg	–	–	–	**35.82**	**1.41–912.59**	**0.03**
	IIg/j	**5.29**	**1.11–25.17**	**0.04**	–	–	–

*Age*							
	Adult	1.00	REF	REF	1.00	REF	REF
	Subadult	2.58	0.58–11.53	0.22	**48.45**	**2.02–1163.33**	**0.02**

*Season*							
	Dry	1.00	REF	REF	–	–	–
	Wet	3.98	0.85–18.74	0.08	–	–	–

Adjusting for age class and stranding season in the northern region, the odds of dying due to *S. neurona* were 5.29 times higher (95 % CI [1.11–25.17], p = 0.04**)** for sea otters infected with the IIg/j genotype than for sea otters infected with other genotypes (Table 2). Adjusted for age in the southern region, the odds of dying due to *S. neurona* were approximately 35.82 times higher (95 % CI [1.41–912.59], p = 0.03) for sea otters infected with the Ib/c/d/gg genotype group relative to sea otters infected with other genotypes. Subadult sea otters in the southern region were significantly more likely to develop fatal *S. neurona* infection than adults. Age was not significantly associated with dying due to *S. neurona* in the northern study region. The observed significant associations of genotype and age (subadult versus adult) with fatal *S. neurona* infection, as well as marginal significance of season in the northern region, remained consistent when models were performed using the total population and regional subpopulations with immature sea otters included (Supplemental Tables 5 and 6).

To examine the effect of parasite genotype on infection outcome, we assessed genotype as both categorical and binary variables in univariable and multivariable models (Table 1, Table 2, Supplemental Tables 4–6). Regional models including the dominant genotype in fatal infections (IIg/j in the north and Ib/c/d/gg in the south) as a binary variable provided similar or enhanced fit relative to versions with categorical genotype variables. In univariable models both with and without immature sea otters included, the IIi and VIu/hh genotypes were more likely to result in non-fatal infections (Table 1, Supplemental Table 5); however, infection with these genotypes was not significantly associated with disease outcome in multivariable models.

## 4 Discussion

By combining previously published and newly generated genotyping data, this study provides novel insight on the role of parasite genotype as well as other host and environmental factors on *S. neurona* infection outcome in southern sea otters, a threatened species that is highly susceptible to infection with this parasite. Results indicate that southern sea otters are infected with diverse *S. neurona* genotypes. Twenty-four unique genotypes were identified in this population compared to 12 strains of *T. gondii* reported in sea otters from the same region (Shapiro et al., 2019; Miller et al., 2023). Genotype diversity was not limited to one infection outcome, as both fatal and non-fatal infections had several different genotypes represented. Only eight genotypes overlapped between both groups, with other genotypes exclusively detected in either fatal or non-fatal infections. Sample sizes were too small for many genotypes to draw statistically significant associations.

For more prevalent genotypes, significant associations with disease outcome were identified with strong regional differences. The IIg/j genotype was significantly associated with fatal infections in the northern study region, and the Ib/c/d/gg genotype was significantly associated with fatal infections in the southern region. It is possible that these fatally associated genotypes may possess features of enhanced virulence compared to other genotypes. Due to limited genome-wide studies, little is known about virulence factors contributing to the pathogenesis of *S. neurona* (Blazejewski et al., 2015). A new *S. neurona* antigen type XIII that appeared to exhibit greater virulence was identified in pinnipeds and cetaceans in the Pacific Northwest (Barbosa et al., 2015). It is possible that SnSAGs, and likely other genes, represent markers of differential virulence among *S. neurona* genotypes. Additional genome-wide studies comparing genotypes from fatal and non-fatal infections are needed to further investigate the genomic basis for potential variability in virulence across genotypes. In-vivo or in-vitro infection models may also be needed in the future to more fully demonstrate differential virulence across genotypes, particularly those associated with fatal infections in sea otters.

Only two *S. neurona* genotypes were found in both the northern and southern study regions. Geographic differences in genotype distribution among sea otters may reflect varying distribution of parasite strains within opossum definitive hosts. Links between *S. neurona* genotypes found in marine intermediate hosts and terrestrial definitive hosts residing in adjacent watersheds have been documented for *S. neurona* infections in southern sea otters and other marine mammals (Rejmanek et al., 2010; O'Byrne et al., 2021). Similar strain distribution patterns between felid definitive hosts and sea otters have also been shown for *T. gondii,* although without such strong geographic differences across strain types as seen with *S. neurona* genotypes (Shapiro et al., 2019). Terrestrial field studies are needed to further characterize the genotypes shed by opossum populations throughout the central California coast and to compare them to *S. neurona* genotypes present in sea otters to elucidate land-to-sea transmission dynamics in different regions across the southern sea otter range.

Sex did not significantly affect disease outcome in sea otters infected with *S. neurona*, similar to what has been reported for *T. gondii* in this host (Shapiro et al., 2019). Several previous epidemiologic studies reported that males were more likely to be infected with *S. neurona* (Johnson et al., 2009; Burgess et al., 2020), and that males were more likely to die from *S. neurona* infections compared to females (Miller et al., 2020). However, these previous studies did not include the effect of parasite genotype on mortality which appears to be a strong predictor of infection outcome based on the results of this study.

Age was an important factor in *S. neurona* infection outcome, with subadult sea otters more likely to die from sarcocystosis compared to adults, similar to what has been previously reported (Miller et al., 2020). Differences among age groups in susceptibility to fatal infections may reflect immunological naivete to this pathogen or decreased immune competence in younger animals compared to adults. Congenital infection with *S. neurona* and *T. gondii* has been previously documented in a sea otter (Shapiro et al., 2016), and the possibility of transplacental transmission of parasites may result in fatal protozoal infections in younger sea otters. All immature sea otters examined in this study died of *S. neurona*-associated meningoencephalitis. However, due to the lack of non-fatal controls for immature animals, statistically supported conclusions cannot be made about the susceptibility of immature sea otters to fatal sarcocystosis.

Sea otters that strand and die during the wet season may be more likely to have fatal *S. neurona* infections. Although significant in univariable models, stranding season had less influence on infection outcome in multivariable models adjusted for genotype and age class. This may reflect a bias in the study population as most animals included in this study stranded during the wet season. Additional cases of *S. neurona*-infected sea otters stranding during the dry season may further clarify the role of season on disease outcome.

The seasonal and temporal association between rainfall-driven runoff events and deaths due to *S. neurona* in sea otters has been well established (Kreuder et al., 2003; Miller et al., 2010, 2020; Shapiro et al., 2012). Heavy rainfall leads to increased surface water runoff into coastal watersheds and ultimately the nearshore marine environment, facilitating transport of infectious sporocysts from terrestrial landscapes where they are shed by opossums into coastal sea otter habitats. In this study we used the established seasonal rainfall–streamflow–mortality relationship to evaluate broader seasonal risk patterns rather than analyzing daily precipitation data. Detailed daily rainfall and runoff data have been previously quantified for both the Monterey and Estero Bay regions (Shapiro et al., 2012), and our adjusted wet season definition builds on this previous study to support comparison across multiple years and sites. Establishing event-level temporal linkages between rainfall, runoff, and protozoal sea otter strandings was outside the scope of the current study, which focused instead on broader seasonal patterns of exposure risk and fatal sarcocystosis. The California wet season also partially overlaps with the period of peak sporocyst shedding by opossums (March to July) (Rejmanek et al., 2009), which may result in larger numbers of sporocysts entering the marine environment during this time. This season also overlaps with the peak period of younger, more susceptible sea otters joining the population (Chinn et al., 2016).

Both the 2004 and 2021 *S. neurona*-associated mortality events occurred during the wet season in Estero Bay, which is located in the southern study region. The occurrence of both mortality events in this region likely accounts for the higher proportion of fatal *S. neurona* infections in the southern region compared to the northern region. Interestingly, each mortality event was associated with either a single genotype or a small group of closely related genotypes. All sea otters from the 2004 event were infected with the Ib/c/d/gg genotype, while those from the 2021 event were infected with the Ia genotype. These findings indicate that large-scale *S. neurona* mortality events in sea otters are likely driven by geographically localized, land-to-sea transfer of one or more pulses of a single, possibly more virulent parasite genotype (or a group of closely related genotypes) into the coastal marine environment, rather than simultaneous introduction of several different genotypes. Protozoa-associated epizootics are rarely reported in free-living wildlife and are especially noteworthy because parasite transmission does not occur between affected sea otters; each infection represents individual exposure to the same pathogen within the same area and time period. The pattern for both outbreaks is suggestive of localized, intensive, point-source exposure of sea otters to the same or closely related, possibly more virulent *S. neurona* genotypes.

In addition to being associated with potentially more virulent genotypes, other factors likely contribute to the occurrence of these *S. neurona*-associated mortality events. Both the 2004 and 2021 events occurred in April after periods of heavy rainfall in San Luis Obispo County in the preceding weeks (Miller et al., 2010; www.cimis.water.ca.gov), which may have led to increased numbers of sporocysts being introduced into the marine environment. Of the 24 cases from these events that were included in this study, 18 (75 %) were immature or subadults suggesting that host factors may also influence which sea otters die during these events. Low levels of domoic acid intoxication and perimortem consumption of razor clams were considered contributing factors in the 2004 event (Miller et al., 2010). These factors have not been investigated for the 2021 event due to limited sampling during the SARS-CoV-2 global pandemic, but it is possible that they may have played a similar role in the latter event. The occurrence of both mortality events in Estero Bay is intriguing and suggests that unique geographic and hydrologic factors in this area or features of the local opossum population may also contribute to the higher occurrence of *S. neurona*-associated sea otter mortality events at this location.

Southern sea otters are commonly co-infected with both *S. neurona* and *T. gondii* (Miller et al., 2020). Because both parasites can cause chronic, latent infections with tissue cysts in various organs (Miller et al., 2018), molecularly confirming the presence of both *T. gondii* and *S. neurona* often requires testing multiple tissues and using parasite-specific PCR assays to selectively detect the DNA of one parasite when the other parasite is in greater abundance. Screening PCR at the ITS1 locus co-amplified both parasites in some of the sea otters included in this study. However, extensive additional testing to definitively determine the *T. gondii* co-infection status for the remaining animals was not performed due to resource limitations. Infection with multiple parasites has been shown to increase disease severity in marine mammals infected with *T. gondii* (Gibson et al., 2011). Future studies would be needed to provide insight on the potential influence of *T. gondii* co-infection on *S. neurona* infection outcome in sea otters.

Several limitations exist for this study. Molecular characterization of non-fatal infections proved challenging and our knowledge of genotypes infecting these animals is limited by sample size. Tongue was the most sensitive tissue for detecting *S. neurona* DNA in non-fatally infected sea otters, similar to the detection of *T. gondii* in terrestrial carnivores (VanWormer et al., 2014). Despite screening tongue tissue for 126 non-fatal infections, a complete genotype could only be determined for 31 (24.6 %) of these sea otters, presumably due to lower numbers of *S. neurona* organisms present in tissues in chronically infected animals that survive compared to those that die from the infection. Additionally, the method used to determine *S. neurona* genotype in this study relies on a series of nested PCR assays that may selectively amplify DNA from a dominant genotype, precluding characterization of mixed genotype infections, if present. For fatal infections, the genotype responsible for disease and death is likely the highest in abundance within affected tissues, and the genotype determined for these cases likely reflects the fatal genotype. For non-fatal infections, the possibility of equal abundance, mixed genotype infections cannot be excluded using current methods. Different sample types were used for genotype characterization across the sea otters included in this study to maximize the likelihood of *S. neurona* DNA amplification for different disease outcomes (e.g., primarily isolates and frozen brain tissue for fatal infections and primarily frozen tongue for non-fatal infections). However, differences in sample type may also have biased the genotypes detectable in different tissue types.

The MLST scheme used in this study utilized six loci that were shown to exhibit the greatest polymorphism across genotypes (Rejmanek et al., 2010; O'Byrne et al., 2021). Other studies have utilized additional loci to further distinguish between genotypes (Wendte et al., 2010a, 2010b; Rejmanek et al., 2010). Limitation of the MLST to six loci necessitated collapsing several microsatellite types together. For future work characterizing additional loci used in other studies could provide greater resolution between closely related genotypes. Additionally, the opportunistic nature of sample collection, the data available for the case selection process, and resource limitations led to an unequal distribution of cases across the years represented in this study (1999–2023) with a gap in cases from 2016 to 2020. This distribution makes it challenging to evaluate shifts in genotype distribution and *S. neurona*-associated mortality rates over the full duration of the study period.

The results of this study show that parasite genotype, in combination with other host and environmental factors, likely influences *S. neurona* infection outcome in southern sea otters. Two genotypes (Ib/c/d/gg and IIg/j) were associated with fatal disease, which exhibited strong geographic patterns. Subadults were more likely to die from sarcocystosis in the southern region, and fatal sarcocystosis may be associated with stranding during the wet season in the northern region. These findings have important implications for the population recovery of this federally protected threatened species. Identification of potential hotspots, such as Estero Bay, for infection by potentially more virulent genotypes and periodic mortality events, can inform decisions regarding locations for sea otter reintroduction and future range expansion efforts. Opossums, endemic to the southeastern United States, were first introduced to California in the early 20th century and have since spread throughout the state. Growing opossum populations and changes in precipitation patterns due to climate change, such as the projected increased occurrence of atmospheric rivers in California (Dettinger, 2011), may exacerbate impacts of *S. neurona*-associated mortality on sea otters. Knowledge of the genotypes and risk factors associated with *S. neurona* mortality may also aid in understanding the epidemiology and pathogenesis of sarcocystosis in other marine mammals that are vulnerable to *S. neurona* infections and suffer from *S. neurona*-induced mortality, such as California sea lions (*Zalophus californianus*), Pacific harbor seals (*Phoca vitulina*), threatened Guadalupe fur seals (*Arctocephalus townsendi*), and Steller sea lions (*Eumetopias jubatus*) (Gibson et al., 2011; Barbosa et al., 2015; Seguel et al., 2019).

## CRediT authorship contribution statement

**Devinn M. Sinnott:** Writing – original draft, Visualization, Validation, Resources, Project administration, Methodology, Investigation, Funding acquisition, Formal analysis, Data curation, Conceptualization. **Melissa A. Miller:** Writing – review & editing, Visualization, Supervision, Resources, Investigation, Funding acquisition, Conceptualization. **Elizabeth VanWormer:** Writing – review & editing, Software, Resources, Methodology, Formal analysis, Data curation. **Francesca Batac:** Writing – review & editing, Investigation, Data curation. **Katherine Greenwald:** Writing – review & editing, Investigation, Data curation. **Colleen Young:** Writing – review & editing, Investigation, Data curation. **Pádraig J. Duignan:** Writing – review & editing, Resources, Investigation. **Margaret E. Martinez:** Writing – review & editing, Resources, Investigation. **Cara L. Field:** Writing – review & editing, Resources. **Michael D. Harris:** Writing – review & editing, Investigation, Data curation. **Heather Harris:** Writing – review & editing, Investigation, Data curation. **Mary E. Gomes:** Writing – review & editing, Investigation, Data curation. **Michael J. Murray:** Writing – review & editing, Resources. **Karen Shapiro:** Writing – review & editing, Supervision, Resources, Project administration, Funding acquisition, Formal analysis, Conceptualization.

## Conflicts of interest

All authors have no conflicts of interest to declare.

## References

[bib1] Asmundsson I.M., Rosenthal B.M. (2006). Isolation and characterization of microsatellite markers from *Sarcocystis neurona*, a causative agent of equine protozoal myeloencephalitis. Mol. Ecol. Notes.

[bib2] Barbosa L., Johnson C.K., Lambourn D.M., Gibson A.K., Haman K.H., Huggins J.L., Sweeny A.R., Sundar N., Raverty S.A., Grigg M.E. (2015). A novel *Sarcocystis neurona* genotype XIII is associated with severe encephalitis in an unexpectedly broad range of marine mammals from the northeastern Pacific Ocean. Int. J. Parasitol..

[bib3] Blazejewski T., Nursimulu N., Pszenny V., Dangoudoubiyam S., Namasivayam S., Chiasson M.A., Chessman K., Tonkin M., Swapna L.S., Hung S.S. (2015). Systems-based analysis of the *Sarcocystis neurona* genome identifies pathways that contribute to a heteroxenous life cycle. mBio.

[bib4] Burgess T.L., Tinker M.T., Miller M.A., Smith W.A., Bodkin J.L., Murray M.J., Nichol L.M., Saarinen J.A., Larson S., Tomoleoni J.A. (2020). Spatial epidemiological patterns suggest mechanisms of land-sea transmission for *Sarcocystis neurona* in a coastal marine mammal. Sci. Rep..

[bib5] Chinn S.M., Miller M.A., Tinker M.T., Staedler M.M., Batac F.I., Dodd E.M., Henkel L.A. (2016). The high cost of motherhood: end-lactation syndrome in southern sea otters (*Enhydra lutris nereis*) on the central California coast, USA. J. Wildl. Dis..

[bib6] Crowdus C.A., Marsh A.E., Saville W.J., Lindsay D.S., Dubey J.P., Granstrom D.E., Howe D.K. (2008). SnSAG5 is an alternative surface antigen of *Sarcocystis neurona* strains that is mutually exclusive to SnSAG1. Vet. Parasitol..

[bib7] Dettinger M. (2011). Climate change, atmospheric rivers, and floods in California - a multimodel analysis of storm frequency and magnitude changes. J American Water Resour Assoc.

[bib8] Gibson A.K., Raverty S., Lambourn D.M., Huggins J., Magargal S.L., Grigg M.E. (2011). Polyparasitism is associated with increased disease severity in *Toxoplasma gondii*-infected marine sentinel species. PLoS Neglected Trop. Dis..

[bib9] Hosmer D., Lemeshow S. (2000).

[bib10] Howe D.K., Gaji R.Y., Marsh A.E., Patil B.A., Saville W.J., Lindsay D.S., Dubey J.P., Granstrom D.E. (2008). Strains of *Sarcocystis neurona* exhibit differences in their surface antigens, including the absence of the major surface antigen SnSAG1. Int. J. Parasitol..

[bib11] Johnson C.K., Tinker M.T., Estes J.A., Conrad P.A., Staedler M., Miller M.A., Jessup D.A., Mazet J.A.K. (2009). Prey choice and habitat use drive sea otter pathogen exposure in a resource-limited coastal system. Proc. Natl. Acad. Sci. U.S.A.

[bib12] Kosmidis I. (2023). brglm2: Bias reduction in generalized Linear models. Comput. Software R.

[bib13] Kreuder C., Miller M.A., Jessup D.A., Lowenstine L.J., Harris M.D., Ames J.A., Carpenter T.E., Conrad P.A., Mazet J.A. (2003). Patterns of mortality in southern sea otters (*Enhydra lutris nereis*) from 1998-2001. J. Wildl. Dis..

[bib14] Melo M.B., Jensen K.D.C., Saeij J.P.J. (2011). *Toxoplasma gondii* effectors are master regulators of the inflammatory response. Trends Parasitol..

[bib15] Milanes C., Kadir T., Lock B., Miller G., Monserrat L., Randles K. (2022).

[bib16] Miller M.A., Gardner I.A., Kreuder C., Paradies D.M., Worcester K.R., Jessup D.A., Dodd E., Harris M.D., Ames J.A., Packham A.E. (2002). Coastal freshwater runoff is a risk factor for *Toxoplasma gondii* infection of southern sea otters (*Enhydra lutris nereis*). Int. J. Parasitol..

[bib17] Miller M.A., Barr B.C., Nordhausen R., James E.R., Magargal S.L., Murray M., Conrad P.A., Toy-Choutka S., Jessup D.A., Grigg M.E. (2009). Ultrastructural and molecular confirmation of the development of *Sarcocystis neurona* tissue cysts in the central nervous system of southern sea otters (*Enhydra lutris nereis*). Int. J. Parasitol..

[bib18] Miller M.A., Conrad P.A., Harris M., Hatfield B., Langlois G., Jessup D.A., Magargal S.L., Packham A.E., Toy-Choutka S., Melli A.C. (2010). A protozoal-associated epizootic impacting marine wildlife: mass-mortality of southern sea otters (*Enhydra lutris nereis*) due to *Sarcocystis neurona* infection. Vet. Parasitol..

[bib19] Miller M.A., Shapiro K., Murray M.J., Haulena M., Raverty S., Gulland F.M.D., Dierauf L.A., Whitman K.L. (2018). CRC Handbook of Marine Mammal Medicine.

[bib20] Miller M.A., Moriarty M.E., Henkel L., Tinker M.T., Burgess T.L., Batac F.I., Dodd E., Young C., Harris M.D., Jessup D.A. (2020). Predators, disease, and environmental change in the nearshore ecosystem: mortality in southern sea otters (*Enhydra lutris nereis*) from 1998–2012. Front. Mar. Sci..

[bib21] Miller M.A., Newberry C.A., Sinnott D.M., Batac F.I., Greenwald K., Reed A., Young C., Harris M.D., Packham A.E., Shapiro K. (2023). Newly detected, virulent *Toxoplasma gondii* COUG strain causing fatal steatitis and toxoplasmosis in southern sea otters (*Enhydra lutris nereis*). Front. Mar. Sci..

[bib22] O'Byrne A.M., Lambourn D.M., Rejmanek D., Haman K., O'Byrne M., VanWormer E., Shapiro K. (2021). *Sarcocystis neurona* transmission from opossums to marine mammals in the Pacific Northwest. EcoHealth.

[bib23] Rejmanek D., Miller M.A., Grigg M.E., Crosbie P.R., Conrad P.A. (2010). Molecular characterization of *Sarcocystis neurona* strains from opossums (*Didelphis virginiana*) and intermediate hosts from central California. Vet. Parasitol..

[bib24] Rejmanek D., Vanwormer E., Miller M.A., Mazet J.A.K., Nichelason A.E., Melli A.C., Packham A.E., Jessup D.A., Conrad P.A. (2009). Prevalence and risk factors associated with *Sarcocystis neurona* infections in opossums (*Didelphis virginiana*) from central California. Vet. Parasitol..

[bib25] R Core Team (2024).

[bib26] Seguel M., Colegrove K.M., Field C., Whoriskey S., Norris T., Duignan P. (2019). Polyphasic rhabdomyositis in California sea lions (*Zalophus californianus*): pathology and potential causes. Vet. Pathol..

[bib27] Shapiro K., Miller M., Mazet J. (2012). Temporal association between land-based runoff events and California sea otter (*Enhydra lutris nereis*) protozoal mortalities. J. Wildl. Dis..

[bib28] Shapiro K., Miller M.A., Packham A.E., Aguilar B., Conrad P., VanWormer E., Murray M.J. (2016). Dual congenital transmission of *Toxoplasma gondii* and *Sarcocystis neurona* in a late-term aborted pup from a chronically infected southern sea otter (*Enhydra lutris nereis*). Parasitology.

[bib29] Shapiro K., VanWormer E., Packham A., Dodd E., Conrad P.A., Miller M. (2019). Type X strains of *Toxoplasma gondii* are virulent for southern sea otters (*Enhydra lutris nereis*) and present in felids from nearby watersheds. Proc. Biol. Sci..

[bib30] Sibley L.D., Boothroyd J.C. (1992). Virulent strains of *Toxoplasma gondii* comprise a single clonal lineage. Nature.

[bib31] Su C., Khan A., Zhou P., Majumdar D., Ajzenberg D., Dardé M.-L., Zhu X.-Q., Ajioka J.W., Rosenthal B.M., Dubey J.P. (2012). Globally diverse *Toxoplasma gondii* isolates comprise six major clades originating from a small number of distinct ancestral lineages. Proc. Natl. Acad. Sci. U.S.A.

[bib32] Thomas N.J., Dubey J.P., Lindsay D.S., Cole R.A., Meteyer C.U. (2007). Protozoal meningoencephalitis in sea otters (*Enhydra lutris*): a histopathological and immunohistochemical study of naturally occurring cases. J. Comp. Pathol..

[bib33] VanWormer E., Miller M.A., Conrad P.A., Grigg M.E., Rejmanek D., Carpenter T.E., Mazet J.A.K. (2014). Using molecular epidemiology to track *Toxoplasma gondii* from terrestrial carnivores to marine hosts: implications for public health and conservation. PLoS Neglected Trop. Dis..

[bib34] Wendte J.M., Miller M.A., Lambourn D.M., Magargal S.L., Jessup D.A., Grigg M.E. (2010). Self-mating in the definitive host potentiates clonal outbreaks of the apicomplexan parasites *Sarcocystis neurona* and *Toxoplasma gondii*. PLoS Genet..

[bib35] Wendte J.M., Miller M.A., Nandra A.K., Peat S.M., Crosbie P.R., Conrad P.A., Grigg M.E. (2010). Limited genetic diversity among *Sarcocystis neurona* strains infecting southern sea otters precludes distinction between marine and terrestrial isolates. Vet. Parasitol..

