# Supplementary material for: Parasite genotype is a risk factor for Sarcocystis neurona-associated mortality in southern sea otters (Enhydra lutris nereis)
Source: Int J Parasitol Parasites Wildl. 2025 Dec 6;29:101174. doi: 10.1016/j.ijppaw.2025.101174 (PMC12741392; doi:10.1016/j.ijppaw.2025.101174)
Supplement: Multimedia component 1 [file mmc1.pdf]

Supplemental Table 1: Primers and cycling conditions for ITS1 and MLST PCR assays.

Primer Name	Primer Sequence (5'-3')	Cycling Conditions	Reference
ITS1 extF	TACCGATTGAGTGTTCCGGTG	(94°C x 3m) + 35[(95°C x 40s) +(58°C x 40s) +(72°C x 90s)] + (72°C x 4m)	Rejmanek et al. 2009
ITS1 extR	GCAATTCACATTGCGTTTCGC		
ITS1 intF	CGTAACAAGGTTTCCGTAGG	(94°C x 3m) + 35[(95°C x 40s) +(59°C x 40s) +(72°C x 90s)] + (72°C x 4m)	
ITS1 intR	TTCATCGTTGCGCGAGCCAAG		
SnSAG1-5-6 extF	TGCTGCATCATTAGGGTCAG	(94°C x 10m) + 35[(95°C x 40s) + (58°C x 40s) + (72°C x 90s)] + (72°C x 10m)	Wendte et al. 2010
SnSAG1-5-6 extR	GCTGTGGGAGTAAGCAGGAT		
SnSAG1-5-6 intF	CGCCAGAGTGTCACTTGTGA		This study
SnSAG1-5-6 intR	CTGGGTAGGGCTTGCTCTTT		
SnSAG3 extF	TCAAGGACGTTTTTCCCTGT	(94°C x 10m) + 35[(95°C x 40s) + (58°C x 40s) + (72°C x 90s)] + (72°C x 5m)	Wendte et al. 2010
SnSAG3 extR	CTCTGCATGCTGCAATGAAT		
SnSAG3 intF	CCCTGCCTTTCTGGTCTCTT		
SnSAG3 intR	TTCTCCCCAAAGACCATCTG		
SnSAG4 extF	ATTAATGCCACGTACTGCTG	(94°C x 10m) + 35[(95°C x 40s) + (58°C x 40s) + (72°C x 90s)] + (72°C x 5m)	Wendte et al. 2010
SnSAG4 extR	TCCACCAATAGTTTAGGCTG		
SnSAG4 intF	ATTGGGAACAGTTTCCATCG		
SnSAG4 intR	CACCTATTCAAATGGCTGTC		
Sn3 extF	CAGGTCGTCCATTTTGGTCT	(94°C x 10m) + 35[(95°C x 40s) + (59°C x 40s) + (72°C x 90s)] + (72°C x 5m)	Asmundsson and Rosenthal 2006
Sn3 extR	GGATAAGGCACGCTTACAGG		
Sn3 intF	CAGCAGGTCGTCCATTTTGG		Rejmanek et al. 2010
Sn3 intR	ACGTGCACGTGCATTGACAC		
Sn7 extF	CGACAGTTCTCCCTGCTCTT	(94°C x 10m) + 35[(95°C x 40s) + (59°C x 40s) + (72°C x 90s)] + (72°C x 5m)	Rejmanek et al. 2010
Sn7 extR	CATGCATCGATTTCTGATCG		
Sn7 intF	CCTGGTGATGAGTGAACTGC		Asmundsson and Rosenthal 2006
Sn7 intR	TGGCCACCAGAGTCTTCTTT		
Sn9 extF	CTGCTGCTAGCGGACTCTCT	(94°C x 10m) + 35[(95°C x 40s) + (59°C x 40s) + (72°C x 90s)] + (72°C x 5m)	Rejmanek et al. 2010
Sn9 intF	CGCCAAAAGACTCACAAACA		Asmundsson and Rosenthal 2006
Sn9 ext/intR	ACGCGCCTAAACGTGAATAG		

Supplemental Table 2: Metadata for the 125 southern sea otters included in univariate and multivariate analyses in this study. Genotype data included characterization performed in previous studies (n=57) and data generated in the present study (n=68). Sea otters that died during the 2004 *S. neurona*-associated mortality event are highlighted in blue and those that died during the 2021 *S. neurona*-associated mortality event are highlighted in green.

ID#	Year	Region	Sample	Genotype	SnSAG1-5-6*	SnSAG3† (239, 503, 504, 1057)	SnSAG4‡ (592)	Sn3 $(AT)^n$	Sn7 $(CA)^n$	Sn9 $(GT)^n$	Infection Outcome	Study
3106	1999	north	isolate	IIi	1	G..T	.	12	17	17	fatal	Wendte et al., 2010b
3339	2000	north	isolate	Vn	5	G..T	.	11	18	16	fatal	Wendte et al., 2010a
3371	2000	north	tongue	Ia	5	.	.	11	22	17	non-fatal	Present study
3483	2001	south	isolate	Ia	5	.	.	11	22	17	non-fatal	Wendte et al., 2010b
3485	2001	south	isolate	Ia	5	.	.	11	22	17	fatal	Wendte et al., 2010b
3501	2001	south	isolate	Ia	5	.	.	11	22	17	fatal	Wendte et al., 2010b
3508	2001	south	isolate	Ib/c/d/gg	5	.	.	11	21	17	fatal	Wendte et al., 2010a
3523	2001	north	isolate	VIIy	6	.	G	11	19	14	fatal	Wendte et al., 2010b
3528	2001	north	isolate	IIg/j	1	G..T	.	12	17	18	fatal	Wendte et al., 2010b
3629	2001	north	isolate	IIg/j	1	G..T	.	12	17	18	fatal	Wendte et al., 2010b
3634	2001	north	isolate	Xw/z	6	.	.	10	20	14	fatal	Wendte et al., 2010a
3639	2001	north	isolate	VIIx	6	.	G	11	20	14	fatal	Wendte et al., 2010b
3660	2002	north	isolate	IIg/j	1	G..T	.	12	17	18	fatal	Wendte et al., 2010a
3866	2003	north	isolate	IIg/j	1	G..T	.	12	17	18	fatal	Wendte et al., 2010b
3892	2003	south	isolate	Ia	5	.	.	11	22	17	fatal	Wendte et al., 2010b
4135	2004	south	isolate	Ib/c/d/gg	5	.	.	11	21	17	fatal	Wendte et al., 2010b
4151	2004	south	isolate	Ib/c/d/gg	5	.	.	11	21	17	fatal	Wendte et al., 2010b
4166	2004	south	isolate	Ib/c/d/gg	5	.	.	11	21	17	fatal	Wendte et al., 2010b
4167	2004	south	isolate	Ib/c/d/gg	5	.	.	11	21	17	fatal	Wendte et al., 2010b
4168	2004	south	isolate	Ib/c/d/gg	5	.	.	11	21	17	fatal	Wendte et al., 2010b
4169	2004	south	isolate	Ib/c/d/gg	5	.	.	11	21	17	fatal	Wendte et al., 2010b
4171	2004	south	isolate	Ib/c/d/gg	5	.	.	11	21	17	fatal	Wendte et al., 2010b
4174	2004	south	isolate	Ib/c/d/gg	5	.	.	11	21	17	fatal	Wendte et al., 2010a
4177	2004	south	isolate	Ib/c/d/gg	5	.	.	11	21	17	fatal	Wendte et al., 2010a
4178	2004	south	isolate	If	5	.	.	11	22	18	fatal	Wendte et al., 2010b
4181	2004	north	isolate	IIg/j	1	G..T	.	12	17	18	fatal	Wendte et al., 2010b
4183	2004	south	isolate	Ib/c/d/gg	5	.	.	11	21	17	fatal	Wendte et al., 2010a

ID#	Year	Region	Sample	Genotype	SnSAG1-5-6*	SnSAG3† (239, 503, 504, 1057)	SnSAG4‡ (592)	Sn3 $(AT)^n$	Sn7 $(CA)^n$	Sn9 $(GT)^n$	Infection Outcome	Study
4189	2004	south	isolate	Ib/c/d/gg	5	.	.	11	21	17	fatal	Wendte et al., 2010a
4194	2004	north	isolate	IXw/z	1	.	.	10	20	14	fatal	Wendte et al., 2010b
4195	2004	south	isolate	Ib/c/d/gg	5	.	.	11	21	17	fatal	Wendte et al., 2010a
4202	2004	south	isolate	Ib/c/d/gg	5	.	.	11	21	17	fatal	Wendte et al., 2010a
4240	2004	north	isolate	Xw/z	6	.	.	10	20	14	fatal	Wendte et al., 2010a
4285	2004	south	isolate	Ib/c/d/gg	5	.	.	11	21	17	fatal	Wendte et al., 2010b
4289	2004	south	isolate	Ib/c/d/gg	5	.	.	11	21	17	fatal	Wendte et al., 2010a
4349	2004	north	tongue	IImm	1	G..T	.	12	18	17	non-fatal	Present study
4387	2005	north	isolate	IIg/j	1	G..T	.	12	17	18	fatal	Wendte et al., 2010a
4413	2005	south	isolate	III l	5	G..T	G	11	18	17	fatal	Wendte et al., 2010a
4529	2005	north	isolate	IIg/j	1	G..T	.	12	17	18	fatal	Wendte et al., 2010a
4530	2005	south	isolate	Ie	5	.	.	11	21	16	fatal	Rejmanek et al., 2010
4653	2006	north	isolate	IIg/j	1	G..T	.	12	17	18	fatal	Rejmanek et al., 2010
4660	2006	south	tongue	Ia	5	.	.	11	22	17	non-fatal	Present study
4663	2006	north	tongue	Xw/z	6	.	.	10	20	14	non-fatal	Present study
4697	2006	north	isolate	IIg/j	1	G..T	.	12	17	18	fatal	Rejmanek et al., 2010
4711	2006	north	isolate	IIg/j	1	G..T	.	12	17	18	fatal	Rejmanek et al., 2010
4724	2006	north	tongue	VIIy	6	.	G	11	19	14	non-fatal	Present study
4725	2006	north	isolate	VIu/hh	5	.	G	10	18	14	fatal	Rejmanek et al., 2010
4742	2006	north	tongue	IIg/j	1	G..T	.	12	17	18	non-fatal	Present study
4743	2006	north	tongue	Ia	5	.	.	11	22	17	non-fatal	Present study
4755	2006	south	isolate	Ia	5	.	.	11	22	17	fatal	Rejmanek et al., 2010
4786	2006	north	isolate	IIg/j	1	G..T	.	12	17	18	fatal	Rejmanek et al., 2010
4834	2006	north	isolate	II/Vh	.	G..T	.	12	16	18	non-fatal	Rejmanek et al., 2010
4848	2006	north	tongue	IIii	1	G..T	.	13	17	17	non-fatal	Present study
4870	2006	south	tongue	IIjj	1	G..T	.	13	17	17	non-fatal	Present study
4928	2007	north	isolate	IIg/j	1	G..T	.	12	17	18	fatal	Rejmanek et al., 2010
4946	2007	north	tongue	VIu/hh	5	.	G	10	18	14	non-fatal	Present study
4970	2007	north	isolate	IIg/j	1	G..T	.	12	17	18	fatal	Rejmanek et al., 2010
4972	2007	south	isolate	VIIy	6	.	G	11	19	14	fatal	Rejmanek et al., 2010
5002	2007	north	isolate	VIIy	6	.	G	11	19	14	fatal	Rejmanek et al., 2010
5011	2007	north	tongue	VIIx	6	.	G	11	20	14	non-fatal	Present study

ID#	Year	Region	Sample	Genotype	SnSAG1-5-6*	SnSAG3† (239, 503, 504, 1057)	SnSAG4‡ (592)	Sn3 $(AT)^n$	Sn7 $(CA)^n$	Sn9 $(GT)^n$	Infection Outcome	Study
5073	2007	north	isolate	IIg/j	1	G..T	.	12	17	18	fatal	Rejmanek et al., 2010
5108	2007	north	tongue	IIi	1	G..T	.	12	17	17	non-fatal	Present study
5110	2007	north	isolate	IIg/j	1	G..T	.	12	17	18	fatal	Rejmanek et al., 2010
5124	2007	north	tongue	IIk	1	G..T	.	13	17	18	non-fatal	Present study
5151	2007	north	tongue	VIu/hh	5	.	G	10	18	14	non-fatal	Present study
5168	2007	north	tongue	IIi	1	G..T	.	12	17	17	non-fatal	Present study
5226	2008	north	isolate	IIIk	5	G..T	G	13	17	18	fatal	Rejmanek et al., 2010
5259	2008	south	isolate	Ia	5	.	.	11	22	17	fatal	Rejmanek et al., 2010
5263	2008	south	isolate	Ib/c/d/gg	5	.	.	11	21	17	fatal	Rejmanek et al., 2010
5274	2008	north	isolate	Ia	5	.	.	11	22	17	fatal	Rejmanek et al., 2010
5278	2008	north	isolate	Ia	5	.	.	11	22	17	fatal	Rejmanek et al., 2010
5283	2008	north	isolate	IVm	.	G--T	.	11	18	17	fatal	Rejmanek et al., 2010
5296	2008	north	isolate	IIg/j	1	G..T	.	12	17	18	fatal	Rejmanek et al., 2010
5418	2008	north	tongue	Ia	5	.	.	11	22	17	non-fatal	Present study
5457	2009	north	tongue	IIi	1	G..T	.	12	17	17	non-fatal	Present study
5501	2009	north	tongue	II II	1	G..T	.	12	16	17	non-fatal	Present study
5558	2009	south	tongue	Ia	5	.	.	11	22	17	non-fatal	Present study
5607	2009	north	tongue	IIi	1	G..T	.	12	17	17	non-fatal	Present study
5670	2009	north	tongue	IIi	1	G..T	.	12	17	17	non-fatal	Present study
5700	2010	north	tongue	VIu/hh	5	.	G	10	18	14	non-fatal	Present study
5824	2010	south	tongue	Ie	5	.	.	11	21	16	non-fatal	Present study
5870	2010	north	tongue	Xw/z	6	.	.	10	20	14	non-fatal	Present study
6023	2011	south	tongue	Ia	5	.	.	11	22	17	non-fatal	Present study
6381	2012	north	tongue	IIg/j	1	G..T	.	12	17	18	non-fatal	Present study
6554	2012	north	tongue	VIu/hh	5	.	G	10	18	14	non-fatal	Present study
6570	2012	north	tongue	IIg/j	1	G..T	.	12	17	18	non-fatal	Present study
6578	2012	north	tongue	IIi	1	G..T	.	12	17	17	non-fatal	Present study
7692	2015	north	tongue	VIIx	6	.	G	11	20	14	non-fatal	Present study
9897	2021	south	brain	Ia	5	.	.	11	22	17	fatal	Present study
9900	2021	south	brain	Ia	5	.	.	11	22	17	fatal	Present study
9902	2021	south	brain	Ia	5	.	.	11	22	17	fatal	Present study
9905	2021	south	brain	Ia	5	.	.	11	22	17	fatal	Present study

ID#	Year	Region	Sample	Genotype	SnSAG1-5-6*	SnSAG3† (239, 503, 504, 1057)	SnSAG4‡ (592)	Sn3 $(AT)^n$	Sn7 $(CA)^n$	Sn9 $(GT)^n$	Infection Outcome	Study
9906	2021	south	brain	Ia	5	.	.	11	22	17	fatal	Present study
9907	2021	south	brain	Ia	5	.	.	11	22	17	fatal	Present study
9911	2021	south	brain	Ia	5	.	.	11	22	17	fatal	Present study
9912	2021	south	brain	Ia	5	.	.	11	22	17	fatal	Present study
9914	2021	south	tongue	Ia	5	.	.	11	22	17	non-fatal	Present study
9919	2021	south	brain	Ia	5	.	.	11	22	17	fatal	Present study
9926	2021	south	brain	Ia	5	.	.	11	22	17	fatal	Present study
9930	2021	south	brain	Ia	5	.	.	11	22	17	fatal	Present study
9936	2021	south	brain	Ia	5	.	.	11	22	17	fatal	Present study
10146	2022	south	brain	Ib/c/d/gg	5	.	.	11	21	17	fatal	Present study
10151	2022	north	isolate	IIg/j	1	G..T	.	12	17	18	fatal	Present study
10152	2022	north	isolate	IIg/j	1	G..T	.	12	17	18	fatal	Present study
10153	2022	south	isolate	Ia	5	.	.	11	22	17	fatal	Present study
10158	2022	south	brain	Ia	5	.	.	11	22	17	fatal	Present study
10160	2022	south	brain	IIbb	1	G..T	.	12	23	14	fatal	Present study
10162	2022	south	isolate	Ia	5	.	.	11	22	17	fatal	Present study
10176	2022	south	brain	Ia	5	.	.	11	22	17	fatal	Present study
10187	2022	south	brain	Ia	5	.	.	11	22	17	fatal	Present study
10240	2022	south	brain	Ia	5	.	.	11	22	17	fatal	Present study
10241	2022	north	brain	Xw/z	6	.	.	10	20	14	fatal	Present study
10250	2022	south	brain	Ia	5	.	.	11	22	17	fatal	Present study
10413	2023	north	isolate	IIp/q	1	G..T	.	11	17	14	fatal	Present study
10416	2023	south	brain	Ia	5	.	.	11	22	17	fatal	Present study
10450	2023	south	brain	Ib/c/d/gg	5	.	.	11	21	17	fatal	Present study
10456	2023	south	brain	Ia	5	.	.	11	22	17	fatal	Present study
10458	2023	south	brain	Ia	5	.	.	11	22	17	fatal	Present study
10460	2023	north	isolate	IIg/j	1	G..T	.	12	17	18	fatal	Present study
10478	2023	south	brain	Ia	5	.	.	11	22	17	fatal	Present study
10490	2023	north	isolate	Xkk	6	.	.	10	19	14	fatal	Present study
10495	2023	south	isolate	Ia	5	.	.	11	22	17	fatal	Present study
10504	2023	north	brain	IIg/j	1	G..T	.	12	17	18	fatal	Present study
10508	2023	south	brain	Ib/c/d/gg	5	.	.	11	21	17	fatal	Present study

ID#	Year	Region	Sample	Genotype	SnSAG1-5-6*	SnSAG3† (239, 503, 504, 1057)	SnSAG4‡ (592)	Sn3 $(AT)^n$	Sn7 $(CA)^n$	Sn9 $(GT)^n$	Infection Outcome	Study
10521	2023	south	fat	Ia	5	.	.	11	22	17	fatal	Present study
10534	2023	north	isolate	Ia	5	.	.	11	22	17	fatal	Present study

*SnSAG1, 5, or 6 based on highest percent identity to references sequences in GenBank.

†Numbers refer to nucleotide position along reference sequence (GenBank GQ851954) where single nucleotide polymorphisms were examined. "." = consensus.

‡ Numbers refer to nucleotide position along reference sequence (GenBank GQ851957) where single nucleotide polymorphisms were examined. "." = consensus.

Supplemental Table 3: Prevalence data for each *S. neurona* genotype, sea otter sex and age class, and stranding season for the total study population and regional (northern and southern) subpopulations.

	Total Study Population (n=125)			Northern Region Population (n=63)			Southern Region Population (n=62)		
	Fatal (% of fatal)	Non-Fatal (% of non-fatal)	Total (% of total)	Fatal (% of fatal)	Non-Fatal (% of non-fatal)	Total (% of total)	Fatal (% of fatal)	Non-Fatal (% of non-fatal)	Total (% of total)
Genotype									
Ia	33 (35.9%)	8 (24.2%)	41 (32.8%)	3 (8.1%)	3 (11.6%)	6 (9.5%)	30 (54.5%)	5 (71.4%)	35 (56.5%)
Ib/c/d/gg	20 (21.6%)	0 (0%)	20 (16%)	0 (0%)	0 (0%)	0 (0%)	20 (36.5%)	0 (0%)	20 (32.3%)
Ie	1 (1.1%)	1 (3%)	2 (1.6%)	0 (0%)	0 (0%)	0 (0%)	1 (1.8%)	1 (14.3%)	2 (3.2%)
If	1 (1.1%)	0 (0%)	1 (0.8%)	0 (0%)	0 (0%)	0 (0%)	1 (1.8%)	0 (0%)	1 (1.6%)
II ll	0 (0%)	1 (3%)	1 (0.8%)	0 (0%)	1 (3.8%)	1 (1.6%)	0 (0%)	0 (0%)	0 (0%)
II/Vh	0 (0%)	1 (3%)	1 (0.8%)	0 (0%)	1 (3.8%)	1 (1.6%)	0 (0%)	0 (0%)	0 (0%)
IIbb	1 (1.1%)	0 (0%)	1 (0.8%)	0 (0%)	0 (0%)	0 (0%)	1 (1.8%)	0 (0%)	1 (1.6%)
IIg/j	20 (21.6%)	3 (9.1%)	23 (18.4%)	20 (54.1%)	3 (11.6%)	23 (36.5%)	0 (0%)	0 (0%)	0 (0%)
IIi	1 (1.1%)	6 (18.2%)	7 (5.6%)	1 (2.7%)	6 (23.1%)	7 (11.1%)	0 (0%)	0 (0%)	0 (0%)
III l	1 (1.1%)	0 (0%)	1 (0.8%)	0 (0%)	0 (0%)	0 (0%)	1 (1.8%)	0 (0%)	1 (1.6%)
IIii	0 (0%)	1 (3%)	1 (0.8%)	0 (0%)	1 (3.8%)	1 (1.6%)	0 (0%)	0 (0%)	0 (0%)
IIIk	1 (1.1%)	0 (0%)	1 (0.8%)	1 (2.7%)	0 (0%)	1 (1.6%)	0 (0%)	0 (0%)	0 (0%)
IIjj	0 (0%)	1 (3%)	1 (0.8%)	0 (0%)	0 (0%)	0 (0%)	0 (0%)	1 (14.3%)	1 (1.6%)
IIk	0 (0%)	1 (3%)	1 (0.8%)	0 (0%)	1 (3.8%)	1 (1.6%)	0 (0%)	0 (0%)	0 (0%)
IImm	0 (0%)	1 (3%)	1 (0.8%)	0 (0%)	1 (3.8%)	1 (1.6%)	0 (0%)	0 (0%)	0 (0%)
IIp/q	1 (1.1%)	0 (0%)	1 (0.8%)	1 (2.7%)	0 (0%)	1 (1.6%)	0 (0%)	0 (0%)	0 (0%)
IVm	1 (1.1%)	0 (0%)	1 (0.8%)	1 (2.7%)	0 (0%)	1 (1.6%)	0 (0%)	0 (0%)	0 (0%)
IXw/z	1 (1.1%)	0 (0%)	1 (0.8%)	1 (2.7%)	0 (0%)	1 (1.6%)	0 (0%)	0 (0%)	0 (0%)
VIIx	1 (1.1%)	2 (6.2%)	3 (2.4%)	1 (2.7%)	2 (7.7%)	3 (4.8%)	0 (0%)	0 (0%)	0 (0%)
VIIy	3 (3.3%)	1 (3%)	4 (3.2%)	2 (5.4%)	1 (3.8%)	3 (4.8%)	1 (1.8%)	0 (0%)	1 (1.6%)
VIu/hh	1 (1.1%)	4 (12.1%)	5 (4%)	1 (2.7%)	4 (15.5%)	5 (7.9%)	0 (0%)	0 (0%)	0 (0%)
Vn	1 (1.1%)	0 (0%)	1 (0.8%)	1 (2.7%)	0 (0%)	1 (1.6%)	0 (0%)	0 (0%)	0 (0%)
Xkk	1 (1.1%)	0 (0%)	1 (0.8%)	1 (2.7%)	0 (0%)	1 (1.6%)	0 (0%)	0 (0%)	0 (0%)
Xw/z	3 (3.3%)	2 (6.2%)	5 (4%)	3 (8.1%)	2 (7.7%)	5 (7.9%)	0 (0%)	0 (0%)	0 (0%)
Total	*92 (100%)*	*33 (100%)*	*125 (100%)*	*37 (100%)*	*26 (100%)*	*63 (100%)*	*55 (100%)*	*7 (100%)*	*62 (100%)*
Sex									
Male	57 (62%)	25 (75.8%)	82 (65.6%)	23 (62.2%)	21 (80.8%)	44 (69.8%)	34 (61.8%)	4 (57.1%)	38 (61.3%)

Female	35 (38%)	8 (24.2%)	43 (34.4%)	14 (37.8%)	5 (19.2%)	19 (30.2%)	21 (38.25)	3 (42.9%)	24 (38.7%)
Total	*92 (100%)*	*33 (100%)*	*125 (100%)*	*37 (100%)*	*26 (100%)*	*63 (100%)*	*55 (100%)*	*7 (100%)*	*62 (100%)*
Age									
Immature	33 (36.3%)	0 (0%)	33 (26.6%)	10 (27.7%)	0 (0%)	10 (16.1%)	23 (41.8%)	0 (0%)	23 (37.1%)
Subadult	36 (39.5%)	4 (12.1%)	40 (32.3%)	15 (41.7%)	4 (15.4%)	19 (30.6%)	21 (38.2%)	0 (0%)	21 (33.9%)
Adult	22 (24.2%)	29 (87.9%)	51 (41.1%)	11 (30.6%)	22 (84.6%)	33 (53.2%)	11 (20%)	7 (100%)	18 (29%)
Total	*91 (100%)*	*33 (100%)*	*124* (100%)*	*36 (100%)*	*26 (100%)*	*62* (100%)*	*55 (100%)*	*7 (100%)*	*62 (100%)*
Season									
Wet	83 (90.2%)	16 (48.5%)	99 (79.2%)	32 (86.5%)	12 (46.2%)	44 (69.8%)	51 (92.7%)	4 (57.1%)	55 (88.7%)
Dry	9 (9.8%)	17 (51.5%)	26 (20.8%)	5 (13.5%)	14 (53.8%)	19 (30.2%)	4 (7.3%)	3 (42.9%)	7 (11.3%)
Total	*92 (100%)*	*33 (100%)*	*125 (100%)*	*37 (100%)*	*26 (100%)*	*63 (100%)*	*55 (100%)*	*7 (100%)*	*62 (100%)*

*Age unknown for one animal

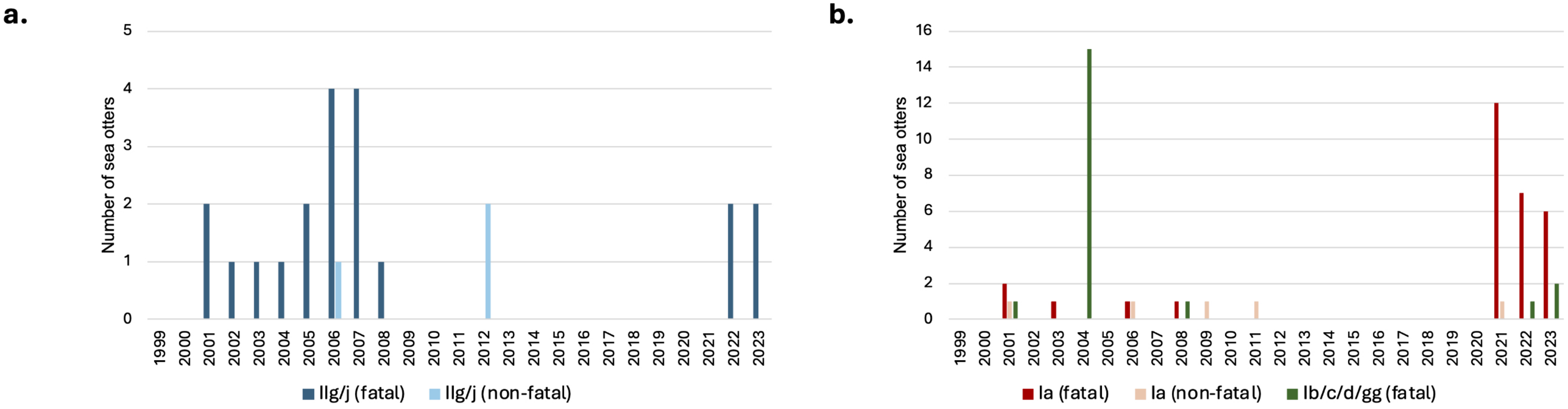

Supplemental Figure 1: Distribution of the most prevalent *Sarcocystis neurona* genotypes characterized from southern sea otters over time in a) the northern region (IIg/j) and b) the southern region (Ia and Ib/c/d/gg).

Supplemental Table 4: Multivariable bias-reduced logistic regression results for associations between fatal *S. neurona* infections and parasite genotype (evaluated as a categorical variable), age class, and stranding season analyzed for the overall study population and each regional subpopulation excluding immature otters. OR, adjusted odds ratio; CI [95%], 95% confidence interval; REF, reference category.

**Variable**	**Level**	**Overall Population (n=91)**			**Northern Population (n=52)**			**Southern Population (n=39)**		
		**OR**	**CI [95%]**	**p-value**	**OR**	**CI [95%]**	**p-value**	**OR**	**CI [95%]**	**p-value**
Genotype										
	Other genotypes	1.00	REF	REF	1.00	REF	REF	1.00	REF	REF
	Ia	1.31	0.32-5.30	0.71	1.93	0.26-14.54	0.52	0.10	0.00-4.95	0.25
	Ib/c/d/gg	**29.34**	**1.37-626.73**	**0.03**	-	-	-	5.98	0.13-282.20	0.36
	IIg/j	2.91	0.54-15.74	0.21	3.91	0.72-21.35	0.12	-	-	-
	VIu/hh	0.33	0.03-4.11	0.39	0.44	0.04-5.00	0.51	-	-	-
	IIi	0.17	0.01-2.02	0.16	0.28	0.03-3.02	0.30	-	-	-
Age										
	Adult	1.00	REF	REF	1.00	REF	REF	1.00	REF	REF
	Subadult	**6.70**	**1.73-26.03**	**<0.01**	3.40	0.67-17.14	0.14	**65.01**	**2.03-2079.10**	**0.02**
Season										
	Dry	1.00	REF	REF	1.00	REF	REF	1.00	REF	REF
	Wet	2.58	0.66-10.17	0.17	3.14	0.65-15.06	0.15	5.39	0.15-188.57	0.35

Supplemental Table 5: Univariable bias-reduced logistic regression results for associations between fatal *S. neurona* infections and parasite (genotype, evaluated as binary and categorical variables), host (age, sex) and environmental (season, geographic region) risk factors analyzed for the entire study population and regional subpopulations with immature otters included. Variables with p-values ≤0.2 were included in further multivariable models (bolded). OR, odds ratio; REF, reference category.

**Variable**	**Level**	**Overall Population (n=125)**		**Northern Population (n=63)**		**Southern Population (n=62)**	
		**OR**	**p-value**	**OR**	**p-value**	**OR**	**p-value**
Genotype Ia							
	Other genotypes	1.00	REF	1.00	REF	1.00	REF
	Ia	1.69	0.25	0.68	0.65	0.54	0.46
Genotype Ib/c/d/gg							
	Other genotypes	1.00	REF	1.00	REF	1.00	REF
	Ib/c/d/gg	**18.94**	**0.05**	-	-	**8.66**	**0.16**
Genotype IIg/j							
	Other genotypes	1.00	REF	1.00	REF	1.00	REF
	IIg/j	**2.46**	**0.15**	**7.87**	**<0.01**	-	-
Genotype VIu/hh							
	Other genotypes	1.00	REF	1.00	REF	1.00	REF
	VIu/hh	**0.11**	**0.03**	**0.21**	**0.14**	-	-
Genotype IIi							
	Other genotypes	1.00	REF	1.00	REF	1.00	REF
	IIi	**0.07**	**0.01**	**0.13**	**0.04**		
Genotype (categorical)							
	Other genotypes	1.00	REF	1.00	REF	1.00	REF
	Ia	**2.82**	**0.06**	0.84	0.85	2.52	0.33
	Ib/c/d/gg	**29.29**	**0.03**	-	-	**18.64**	**0.08**
	IIg/j	**4.18**	**0.04**	**4.92**	**0.03**	-	-
	VIu/hh	**0.23**	**0.19**	0.28	0.26	-	-

	Ili	**0.16**	**0.08**	**0.19**	**0.12**	-	-
Sex							
	Male	1.00	REF	1.00	REF	1.00	REF
	Female	**1.85**	**0.18**	**2.41**	**0.14**	0.80	0.78
Age							
	Adult	1.00	REF	1.00	REF	1.00	REF
	Subadult	**10.63**	**<0.01**	**6.74**	**<0.01**	**28.04**	**0.03**
	Immature	**87.84**	**<0.01**	**41.09**	**0.02**	**30.65**	**0.03**
Season							
	Dry	1.00	REF	1.00	REF	1.00	REF
	Wet	**9.32**	**<0.01**	**6.85**	**<0.01**	**8.90**	**0.02**
Geographic region							
	Northern	1.00	REF	-	-	-	-
	Southern	**5.23**	**<0.01**	-	-	-	-

Supplemental Table 6: Multivariate bias-reduced logistic regression results for associations between fatal *S. neurona* infections and parasite (genotype), host (age) and environmental (season) risk factors for the total study population and regional (northern, southern) subpopulations including immature otters. Genotype was evaluated as a categorical variable across all populations and was further evaluated as a binary variable in models for regional subpopulations. OR, odds ratio; CI [95%], 95% confidence interval; REF, reference category.

**Variable**	**Level**	**Total Population (n=124)**			**Northern Population (n=62)**			**Southern Population (n=62)**		
		**OR**	**CI [95%]**	**p-value**	**OR**	**CI [95%]**	**p-value**	**OR**	**CI [95%]**	**p-value**
Multivariate model with Genotype as categorical variable										
Genotype (categorical)										
	Other genotypes	1.00	REF	REF	1.00	REF	REF	1.00	REF	REF
	Ia	1.31	0.33-5.14	0.70	1.92	0.26-14.35	0.53	0.09	0.00-3.61	0.20
	Ib/c/d/gg	**29.38**	**1.39-622.57**	**0.03**	-	-	-	5.95	0.13-273.51	0.36
	IIg/j	2.91	0.55-15.30	0.21	3.86	0.75-19.81	0.11	-	-	-
	VIu/hh	0.33	0.03-4.10	0.39	0.44	0.04-4.94	0.51	-	-	-
	IIi	0.17	0.01-2.01	0.16	0.28	0.03-2.98	0.29	-	-	-
Age										
	Adult	1.00	REF	REF	1.00	REF	REF	1.00	REF	REF
	Immature	**58.14**	**3.43-986.41**	**<0.01**	13.70	0.60-313.62	0.10	**114.98**	**4.09-3232.15**	**<0.01**

	Subadult	**6.72**	**1.73-26.06**	**<0.01**	3.41	0.68-17.14	0.14	**65.24**	**2.03-2095.41**	**0.02**
Season										
	Dry	1.00	REF	REF	1.00	REF	REF	1.00	REF	REF
	Wet	2.59	0.67-10.01	0.17	3.15	0.67-14.75	0.15	6.20	0.26-146.39	0.26
Multivariate model with Genotype as binary variable										
Genotype (binary)										
	Other genotypes	-	-	-	1.00	REF	REF	1.00	REF	REF
	Ib/c/d/gg	-	-	-	-	-	-	**27.95**	**1.21-647.46**	**0.04**
	IIg/j	-	-	-	**5.22**	**1.15-23.77**	**0.03**	-	-	-
Age										
	Adult	-	-	-	1.00	REF	REF	1.00	REF	REF
	Immature	-	-	-	13.89	0.61-317.46	0.10	**72.67**	**3.01-1754.93**	**<0.01**
	Subadult	-	-	-	2.59	0.58-11.52	0.21	**39.27**	**1.48-1038.64**	**0.03**
Season										
	Dry	-	-	-	1.00	REF	REF	1.00	REF	REF
	Wet	-	-	-	3.99	0.87-18.39	0.08	1.43	0.12-17.52	0.78
